# Poor physical recovery after critical illness: incidence, features, risk factors, pathophysiology, and evidence-based therapies

**DOI:** 10.1097/MCC.0000000000000955

**Published:** 2022-07-05

**Authors:** Yente Florine Niké Boelens, Max Melchers, Arthur Raymond Hubert van Zanten

**Affiliations:** a Department of Intensive Care Medicine, Gelderse Vallei Hospital, Ede, The Netherlands; b Wageningen University & Research, Division of Human Nutrition and Health, Wageningen, The Netherlands

**Keywords:** critical illness, intensive care unit acquired weakness, intensive care unit, muscle weakness, rehabilitation

## Abstract

To summarize the incidence, features, pathogenesis, risk factors, and evidence-based therapies of prolonged intensive care unit (ICU) acquired weakness (ICU-AW). We aim to provide an updated overview on aspects of poor physical recovery following critical illness.

**Recent findings:**

New physical problems after ICU survival, such as muscle weakness, weakened condition, and reduced exercise capacity, are the most frequently encountered limitations of patients with postintensive care syndrome. Disabilities may persist for months to years and frequently do not fully recover. Hormonal and mitochondrial disturbances, impaired muscle regeneration due to injured satellite cells and epigenetic differences may be involved in sustained ICU-AW. Although demographics and ICU treatment factors appear essential determinants for physical recovery, pre-ICU health status is also crucial. Currently, no effective treatments are available. Early mobilization in the ICU may improve physical outcomes at ICU-discharge, but there is no evidence for benefit on long-term physical recovery.

**Summary:**

Impaired physical recovery is observed frequently among ICU survivors. The pre-ICU health status, demographic, and ICU treatment factors appear to be important determinants for physical convalescence during the post-ICU phase. The pathophysiological mechanisms involved are poorly understood, thereby resulting in exiguous evidence-based treatment strategies to date.

## INTRODUCTION

Significant developments in intensive care medicine have improved survival rates, resulting in more intensive care unit (ICU) survivors [1]. However, surviving critical illness may cause new disabilities as part of the ’postintensive care syndrome’ (PICS), comprising physical, mental and cognitive health problems [2].

This review focuses on long-term physical outcomes following critical illness. Long-term muscle weakness after ICU admission, also known as sustained ICU-acquired weakness (ICU-AW), is frequently observed and encompasses critical illness myopathy (CIM), critical illness polyneuropathy (CIP) or a combination [3^▪^,^4^^▪▪^]. Typically, a Medical Research Council (MRC)-sum score <48 is the clinical diagnosis for ICU-AW [5]. ICU patients can lose >15% of their muscle mass within the first week of ICU treatment [6]. Consequently, reduced muscle mass and function impair the ability to perform instrumental activities of daily living and limit the chance of returning to work [7]. Hospital discharge to rehabilitation facilities or nursing homes is necessary in severe cases. Furthermore, in severe ICU-AW (MRC-sum <36), increased mortality up to 1 year after ICU admission has been observed [8].

This review provides an updated overview of aspects of poor physical recovery after critical illness, covering its incidence, features, risk factors, pathophysiology, and evidence-based therapies. 

**Box 1:**
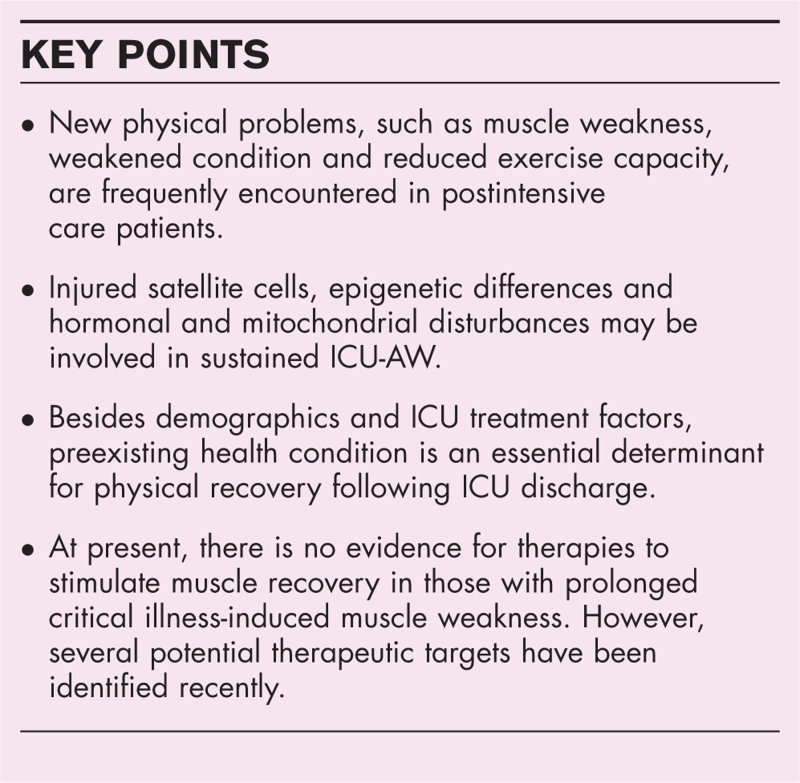
no caption available

## INCIDENCE OF POOR PHYSICAL RECOVERY AND ITS FEATURES

**FIGURE 1:**
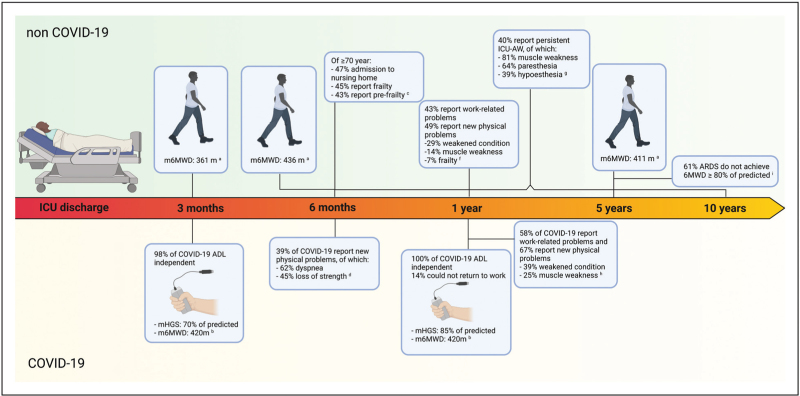
Visualized summary of study results regarding prevalence and incidence of impaired physical function following ICU-discharge in COVID-19 and non-COVID-19 patients. Legend: ^a^ Systematic review and meta-analysis including 16 studies and 1755 ICU survivors [12^▪▪^]; ^b^ Prospective single-center study including 114 COVID-19 ICU survivors, of which 98 analyzed at 3 months, and 51 analyzed at one year [17^▪^]; ^c^ Prospective cohort study including 266 ≥ 70-year-old ICU-survivors [13]; ^d^ Prospective multicenter cohort study including 212 COVID-19 ICU-survivors [15^▪^]; ^e^ Population-based cohort study including 546 ≥ 70-year-old ICU-survivors [33]; ^f^ Prospective multicenter cohort study including 2345 adult ICU-survivors [4^▪▪^]; ^g^ Prospective multicenter cohort study including 301 COVID-19 ICU survivors, of which 246 analyzed at one year [14^▪▪^]; ^h^ Single-center cohort study including 149 ICU-survivors [10^▪^]; ^i^ Single-center cohort study including 109 ARDS ICU-survivors, of which 94 were analyzed at 5 years [11]. 6MWD, Six Minute Walking Distance; ADL, Activities of Daily Living; COVID-19, Coronavirus Disease 2019; ICU, intensive care unit; ICU-AW, ICU-acquired weakness; m6MWD, Mean Six Minute Walking Distance; mHGS, mean Hand Grip Strength. Created with BioRender.com.

Poor physical outcome is frequently encountered in ICU survivors (Fig. 1) [9^▪▪^]. The prevalence of CIP and a combination with CIM is encountered more frequently following ICU discharge than CIM alone [3^▪^]. Patients with CIM only are more likely to recover than patients with CIM and CIP [3^▪^,10^▪^]. Herridge and coworkers showed that exercise capacity five years after ICU-discharge is still below predicted performance in most acute respiratory stress syndrome (ARDS) survivors [11]. In another study, 40% reported persistent ICU-AW symptoms during 6-months to 10-year follow-up [10^▪^]. Remarkably, only 38% recovered, and 62% had persistent symptoms among those patients with ICU-AW at ICU discharge. Additionally, only 11.6% of the cohort was diagnosed with ICU-AW at ICU discharge by physicians, whereas 75% self-reported to have experienced ICU-AW symptoms following ICU-discharge, which stresses the need for ICU-AW awareness among treating physicians and adequate diagnostic procedures during follow-up after discharge [10^▪^].

A recent systematic review found that the mean 6-min-walking-distance-test (6MWD) in survivors remained below population norms following ICU-discharge [12^▪▪^]. A significant increase in mean 6MWD at 12 months compared to 3 months was observed, yet no difference between 12 and 60 months, indicative of stagnation of physical recovery at one year after ICU-discharge.

Notably, previous studies did not consider the prevalence of preexisting impairments. These should be taken into account to prevent overestimating the effects of critical illness on physical function [13]. However, pre-ICU functional performance is not always evaluated. Few published studies investigate new-onset physical problems following ICU discharge.

A sub-study of the MONITOR-IC study concluded that new physical domain problems of PICS were the most symptoms reported one year after ICU discharge and are present in half of the patients [4^▪▪^]. Although possibly associated with other than the physical PICS domain, fatigue was the most reported new symptom. Weakened condition and muscle weakness were the subsequent most frequently encountered physical symptoms after one year [4^▪▪^]. Similar symptoms were reported in another sub-study with post-ICU patients treated for Coronavirus disease 2019 (COVID-19). Two-thirds reported one or more new physical problems one year after ICU treatment [14^▪▪^]. Hodgson and coworkers also evaluated ICU patients after severe COVID-19 [15^▪^]. In total, 71.3% reported persistent symptoms, of which 38.9% experienced new disabilities [15^▪^].

### Coronavirus disease 2019 versus non-Coronavirus disease 2019 patients

COVID-19 ICU patients may be more vulnerable to ICU-AW than non-COVID-19 ICU patients due to a longer duration of mechanical ventilation and ICU stay [14^▪▪^]. Moonen *et al.* report that physical function at ICU discharge is more limited in COVID-19 ICU survivors than non-COVID-19 ICU survivors [16^▪^]. However, muscle function improved more promptly during hospital stay among COVID-19 versus non-COVID-19 ICU survivors, leading to similar functionality at hospital discharge [16^▪^]. When comparing new physical disabilities one year after ICU discharge in non-COVID-19 and COVID-19 patients, incidence rates are higher in the latter. Convalescence in the long term appears not necessary sooner [4^▪▪^,^14^^▪▪^]. Additionally, in COVID-19 ICU survivors, significant improvements in handgrip strength (HGS) but not 6MWD were seen over time [17^▪^]. COVID-19 ICU survivors may experience impaired pulmonary diffusion capacity, resulting in decreased long-term exercise capacity rather than muscle dysfunction [15^▪^,^18^]. Although COVID-19's clinical features differ from classical ARDS, physical function recovery over time was worse in ARDS than in non-ARDS post-ICU patients [12^▪▪^]. Still, 86% of COVID-19 patients could return to work one year after ICU discharge, which is substantially higher than patients surviving classical ARDS (40%) [17^▪^].

## PATHOGENESIS

Reduced muscle strength and weakness are mainly the results of an imbalance between synthesis and lysis of muscle proteins, leading to substantial muscle wasting and maintaining a catabolic state [6,19].

### Acute phase

**FIGURE 2:**
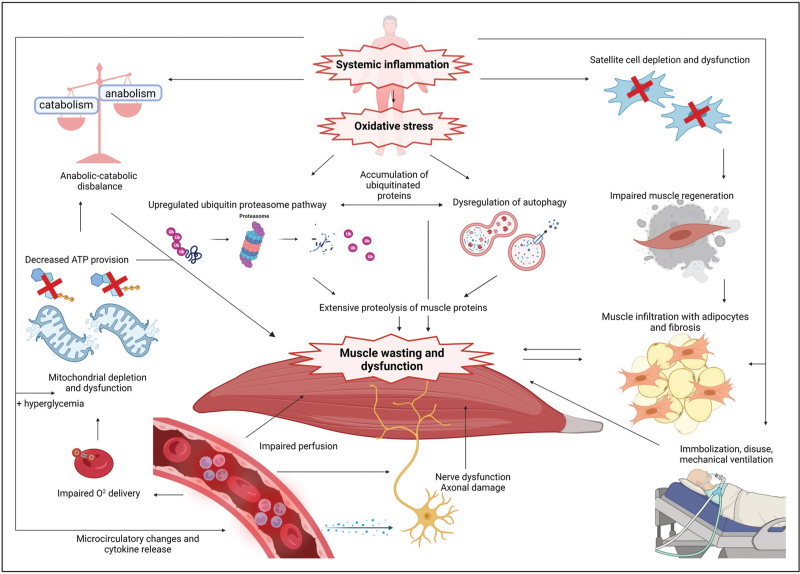
Heading: Pathophysiologic mechanisms possibly involved in developing acute muscle wasting and dysfunction during critical illness, resulting in ICU-acquired weakness. ATP, adenosine triphosphate; O^2^, oxygen. Created with BioRender.com.

Several potential pathophysiologic mechanisms involved in muscle atrophy and dysfunction have been associated with developing ICU-AW (Fig. 2). Systemic inflammation and enhanced oxidative stress during critical illness results in upregulation of ubiquitin-proteolysis pathways and dysregulation of autophagy, which causes rapid and severe muscle proteolysis [19,20]. Inflammation and hyperglycemia also induce loss of mitochondrial capacity and function, compromising energy production, resulting in muscle dysfunction and atrophy [19–21]. Furthermore, peripheral insulin resistance, disturbance of intracellular calcium homeostasis, lipid toxicity, muscle disuse, microcirculatory disturbances and cytokine release resulting in muscle denervation contribute to muscle weakness in the critically ill patient [19–21]. Recently, two less methylated epigenome regions, HIC1 and NADK2, were found in muscle tissue from ICU patients compared with controls [22^▪^]. HIC1 regulates muscle regeneration and moderates the concentration of postsynaptic acetylcholine receptors, whereas NADK2 is involved in fatty lipid metabolism and mitochondrial stimulation [22^▪^].

### Post-intensive care unit phase

**FIGURE 3:**
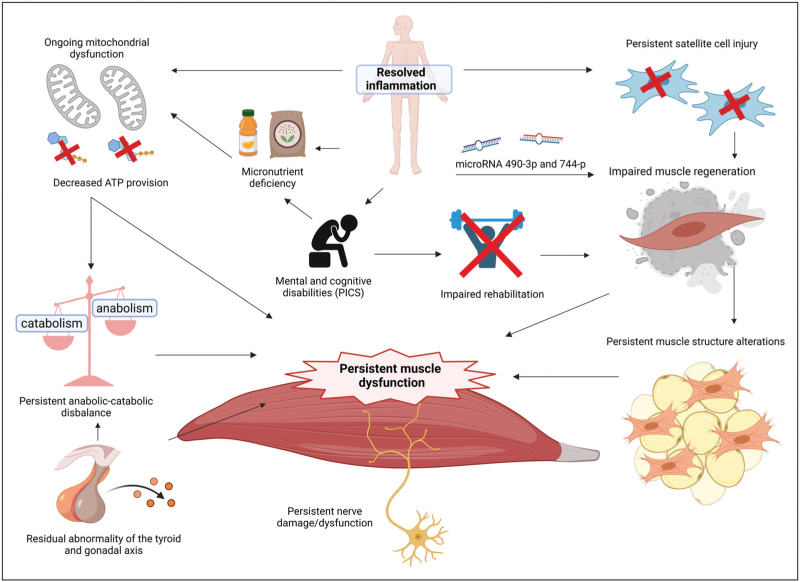
Heading: Pathophysiologic mechanisms possibly involved in prolonged muscle dysfunction following critical illness. ATP, adenosine triphosphate; HIC1, Hypermethylated in Cancer 1; NADK 2, Nicotinamide-adenine-dinucleotide Kinase 2; O2, oxygen; PICS, postintensive care syndrome. Created with BioRender.com.

Although the pathophysiology of muscle atrophy and dysfunction during critical illness seems to be partially elucidated, the mechanisms involved in persistent muscle dysfunction resulting in impaired physical recovery after ICU discharge remain poorly understood (Fig. 3) [20]. Dos Santos and coworkers report that mechanisms involved in acute critical illness-related muscle wasting, i.e., mitochondrial content, enhanced muscle proteolysis, and autophagy dysregulation, have normalized to baseline six months after ICU-admission [23]. Instead, sustained muscle weakness may be explained by persistently impaired muscle regeneration due to decreased numbers of muscle stem cells, better known as satellite cells [23,24]. During critical illness, these cells become depleted due to inflammation and appear to regenerate less in those patients with persistent muscle weakness despite resolved inflammation, indicating long-lasting injury to these cells [23]. Alterations of the epigenome of satellite cells are found in older individuals and those suffering from muscle pathology, reducing the capacity of these cells to regenerate [25]. Modification of these alterations may provide future therapeutic targets for sustained ICU-AW.

Remarkably, the muscle mass of critical illness survivors suffering from sustained muscle weakness appears to normalize whereas contractility remains impaired months after ICU-discharge, suggesting an imbalance between muscle volume and strength [23,26]. Extensive inflammation during critical illness is responsible for alterations in the muscle structure, replacing muscle tissue with adipocytes or fibrosis [19,21]. This fatty infiltration persisted for months in a cohort of post-ICU patients with poor physical recovery [27]. In the same sample, muscle microRNA 490-3p and microRNA-744-p, both negative myogenesis regulators, were increased, potentially impacting the long-term muscle weakness of ICU survivors [28]. Even though these findings may represent some population variability, muscle microRNA regulation may serve as a potential treatment strategy to prevent dysregulated muscle regeneration.

The role of myostatin in musculoskeletal muscle wasting is recently gaining attention [6,29]. Myostatin is a negative regulator of myogenesis and is inversely correlated with inflammation in critically ill patients, possibly preventing excessive muscle wasting in the acute phase [29]. Controversially, complete myostatin inhibition prevents muscle wasting and improves survival in septic mouse models, providing a potential pharmacological target to attenuate critical illness-induced muscle atrophy [30^▪^]. Myostatin was upregulated in patients 9–12 months after surviving major thermal trauma and inversely correlated with muscle strength [31]. However, these findings require confirmation in a general post-ICU population.

Additionally, neuroendocrine alterations in patients with sustained muscle weakness are associated with decreased physical recovery. A higher reverse T3, and lower T3-reverse T3 ratio was found to be associated with lower HGS and shorter 6MWD five years after critical illness [32^▪^]. Persisting abnormalities within the thyroid axis may increase the risk of long-term physical impairment. Nevertheless, the mechanisms involved remain unclear.

## RISK FACTORS BEFORE, DURING AND AFTER INTENSIVE CARE UNIT ADMISSION

Females and the elderly are more prone to poor functional recovery after critical illness [4^▪▪^,10^▪^,^33^]. A reduced ability to stimulate muscle protein synthesis during aging, known as anabolic resistance, develops [34]. Although younger patients may recover faster than the elderly, they do not always recover completely to their pre-ICU health status [11]. Differences between sexes remain unclear but may be associated with smaller preexistent muscle mass in women [5]. However, these patients generally also have a poorer pre-ICU health status [35]. Patients experiencing cognitive, mental or physical health issues before ICU admission are at risk for poor functional outcomes after ICU [4^▪▪^,15^▪^,^34^]. Lastly, patients with middle education are less likely to become frail than patients with low education one year after ICU admission [4^▪▪^].

Several risk factors associated with developing ICU-AW are multiple organ failure, immobilization, hyperglycemia and systemic inflammation [9^▪▪^]. Additionally, the severity of illness and duration of mechanical ventilation are essential predictors of physical outcomes after critical illness [15^▪^,^21^]. Emergency surgery ICU patients and, to a lesser extent, medical ICU patients report significant deteriorations in physical function and quality of life one year after admission, whereas elective surgical patients may experience an improvement [4^▪▪^]. Elective surgical patients typically require short-term ICU admission, whereas other patients are more exposed to systemic inflammation and prolonged organ support [4^▪▪^]. Furthermore, corticosteroid treatment has been associated with ICU-AW development, particularly in mechanically ventilated patients [36]. Although corticosteroids attenuate systemic inflammation and improve hypoxemia, they induce catabolic effects on skeletal muscles [9^▪▪^,^12^^▪▪^]. Additionally, neuromuscular blocking treatment (NMBA) may be associated with muscle weakness as it causes complete muscle disuse, potentially attributing to ICU-AW. However, current guidelines do not recommend against prolonged therapy with NMBA as its effect on muscle wasting has not been confirmed [12^▪▪^,^37^].

Muscle strength at ICU discharge, measured by MRC-sum score, is a predictor for HGS, 6MWD and self-reported physical functioning at five years follow-up [38]. ICU length of stay (LOS) is a significant determinant for one-year physical outcomes; hospital LOS may also contribute to new-onset frailty [4^▪▪^,^33^].

## EVIDENCE-BASED THERAPIES

Currently, no effective treatments are available, and physicians mainly focus on preventing ICU-AW by early mobilization and avoiding hyperglycemia [21,39].

### Early mobilization

Several systematic reviews have been published addressing the effect of early mobilization during ICU admission on outcomes [40–43]. Results are inconsistent comparing these meta-analyses, possibly due to different definitions of early mobilization and inclusion of heterogeneous trials. The most recent and comprehensive meta-analysis, including sixty trials, found that early mobilization during ICU admission improved physical function at hospital discharge [44]. However, beneficial effects on physical function six months after discharge were not found in patients rehabilitated during ICU stay [42,44]. Additionally, evidence of rehabilitation initiated after ICU discharge is insufficient to prove its effect on functional recovery [45]. These findings suggest ongoing mechanisms, such as a persistently disturbing catabolism/anabolism balance, causing the inability to regain muscle function after discharge [23].

### Hormonal therapies

Randomised controlled trials (RCT) studying growth hormone in ICU-patients failed to improve outcomes and even increased mortality [19]. However, the timing of treatment may be crucial as hormonal supplementation may act pro-inflammatory during critical illness. A different approach to influencing the gonadal axis would be through testosterone, as low testosterone levels may persist after discharge [46^▪^]. Treatment with the testosterone analog oxandrolone is beneficial in patients with severe burns to increase lean body mass during recovery [47]. Two small trials with oxandrolone in nonburn ICU-patients could not find an association with improved outcomes but did not focus on the long-term [48,49]. Both groups received a low nutritional intake implicating inadequate substrate administration for muscle regeneration. Moreover, oxandrolone was discontinued during ward stay, which would be the crucial therapeutic phase to regain muscle function for patients with sustained ICU-AW. Therefore, an RCT with testosterone analog in post-ICU patients is warranted to determine the effect on physical rehabilitation [46^▪^].

### Nutritional therapy

A different approach would be through nutritional therapy to restore the anabolic state and stimulate muscle regeneration [50]. Nevertheless, the beneficial effects of elevated protein intake have not yet been shown. In an RCT, personalized nutritional goals were estimated to cover 100% of the requirements in one group, whereas the other group received standard nutrition [51]. Although protein provision was higher in the first group, no difference in self-reported physical function was seen in patients six months after critical illness. Remarkably, early parenteral nutrition (PN) (<48 h) compared to late PN (≥ 8days) was found to increase muscle weakness, but not muscle wasting, during ICU stay [52]. The authors attributed this to more efficient autophagy control among patients who received late PN. Additionally, infusion of ketone bodies or ketogenesis in the acute phase of critical illness attenuated muscle weakness in a murine sepsis model [53]. Ketones can provide energy in conditions of mitochondrial dysfunction. However, this finding requires further exploration in human studies. After 4–7 days of ICU admission, adequate protein and energy intakes are needed to prevent loss of muscle mass and function [50,54].

These nutritional requirements are often not achieved in the post-ICU period, negatively affecting long-term outcomes [55,56]. Research focusing on optimal nutrition strategies during the post-ICU phase is lacking. A recent RCT in non-ICU patients showed that protein supplementation in hospitalized elderly with sarcopenia improved physical performance, reducing rehabilitation time and hospital LOS [57^▪^]. Although these patients differ from ICU patients as the latter may suffer from inflammation and oxidative stress, protein supplementation may still provide an anabolic signal. It could be a potential treatment strategy in patients suffering from muscle wasting during the post-ICU phase, yet it requires further research.

In contrast to Dos Santos *et al.*, mitochondrial damage was found in animals surviving sepsis to persist in those with sustained muscle weakness despite resolved inflammation [23,26]. Moreover, Dos Santos and colleagues did not investigate the actual mitochondrial function, which may contribute to sustained ICU-AW [23]. Consequently, mitochondrial resuscitation with micronutrients after ICU discharge may be interesting, as many vitamins and trace elements are involved in the optimal functioning of mitochondria [58]. High dosages of single micronutrients failed to show benefits. Therefore, a strategic antioxidant cocktail may be more clinically efficient [59^▪^].

### Mesenchymal cells

Considering persistent satellite cell injury as a pivotal driver of poor muscle regeneration, targeting these cells may be a valuable treatment target [23]. Their functionality may be restored by mesenchymal stem cells. In septic murine models, these cells improved muscle recovery, decreased necrosis and fibrosis, and decreased plasma levels of pro-inflammatory cytokines and procalcitonin [24]. No human studies have been published involving mesenchymal cell therapy to regain muscle function in post-ICU patients.

## CONCLUSION

Poor physical recovery is among the most debilitating and reported aspects of PICS among ICU survivors. As most studies do not consider the preexisting health condition when addressing new physical symptoms, the actual incidence of new disabilities is not precisely known. The pre-ICU health status, demographic, and ICU treatment factors appear to be important determinants for physical convalescence post-ICU. The pathophysiological mechanisms involved are poorly understood, thereby resulting in exiguous evidence-based treatment strategies to date. More research into the pathophysiology and promising multimodal therapies involving nutritional, exercise rehabilitation, and hormonal therapies in the post-ICU period are warranted.

## Acknowledgements

*None.*

### Financial support and sponsorship

*None.*

### Conflicts of interest

*A.R.H.V.Z. reported receiving honoraria for advisory board meetings, lectures, research, and travel expenses from AoP Pharma, Baxter, Cardinal Health, Danone-Nutricia, Dim-3, Fresenius Kabi, GE Healthcare, Mermaid, Lyric, and Rousselot. The other authors have nothing to declare.*

## REFERENCES AND RECOMMENDED READING

Papers of particular interest, published within the annual period of review, have been highlighted as:

▪ of special interest
▪▪ of outstanding interest
